# Ophthalmology

**Published:** 1891-03

**Authors:** A. A. Hubbell

**Affiliations:** Professor of Ophthalmology etc., Niagara University


### Progress in Medical Science.

### OPHTHALMOLOGY.

CONDUCTED BY

A. A. HUBBELL, M. D.,

Professor of Ophthalmology etc., Niagara University.

### SOME POINTS OF CONNECTION BETWEEN THE EYE AND THE CARDIO-VASCULAR SYSTEM.

IN A PAPER recently read before the Harverian Society of London, (*British Medical Journal*, November 15, 1890,) Dr. Batten endeavors to show that hypermetropia and myopia are associated with other structural conditions, especially in the cardio-vascular systems, and that, consequently, hypermetropes and myopes are liable to be differently affected by disease. Optic neuritis, for example, is of extreme rarity in myopia, while its occurrence in hypermetrophia is very common, and this he considers not due to local causes only, but to general conditions. In the young hypermetrope, the pulse is quick, easily accelerated, and varies rapidly, but not apparently so much with the degree of manifest, as with latent, hypermetrophia. In high hypermetrophia, with bad vision, the pulse is often a markedly slow one. The tension is low. The heart apex is often inside its normal position, and the first sound of the heart is generally accentuated. Murmurs, of a variable and functional character, frequently occur. Loud atypical systolic murmurs and heart-sounds, resembling those found in chorea, are also met with. In elderly hypermetropes, he considers high-tension pulse and cardiac intermission and irregularity of frequent occurrence. The action of atropine (in the eye.—H.) in slowing the quick hypermetropic pulse, is very marked, and correction by glasses often had the same effect. In myopia, the pulse is generally slow, except in progressive cases, when it is usually rapid. There is accentuation of the second heart-sound, and the tension of the pulse is raised. He regards myopia as a kind of rickets, depending upon a constitutional cause, which affects also the cardio-vascular system. He urges that in the treatment of myopia, sufficient importance is not attached to the constitutional portion of the disease. He concludes: (1.) There is an intimate connection between the eye, in its muscular, nervous and vascular structure, and the cardio-vascular system, both in health and disease. (2.) As regards the hypermetropic form of circulation, partly structural and partly functional, this form of circulation is liable to influence the type of disease in the individual, and gives rise to cardiac irritation and functional disease, leading to malnutrition. (3.) As regards myopia, the changes which lead to it produce also a change in the circulation, partly structural, partly functional, and that the form of circulation, so produced, appears to render the individual less liable to some, and more liable to other, forms of disease.

---

### ARTHRITIS, FOLLOWING PURULENT CONJUNCTIVITIS.

Deutschmann (*Arch. f. Ophthal.*, xxxvi., part i.,) reports two cases of arthritis, following conjunctivitis neonatorum. He also refers to several cases reported by other observers. In all cases in which any statement was made, the third week was given as the time at which the joint affection appeared. The similarity, in clinical aspects, between these cases of arthritis, following conjunctivitis neonatorum, and that which occurs in the course of gonorrhea, is manifest and strongly indicative of the pathogenetic identity of the two inflammations.—*Oph. Rev.*, Nov., 1890.

---

### TREATMENT OF IMMATURE CATARACT BY MANIPULATION, CONJOINED WITH INSTILLATION.

Dr. R. Kalish, of New York, proposes a new method of treating immature cataract (*Medical Record*, Mar. 29th, and Dec. 20, 1890). The instillation consists of equal parts of a one per cent. solution of boracic acid in rose water and glycerine. The proportion of glycerine is diminished, if its use causes too much irritation, lasting five to twenty seconds, or increased to double the amount of boracic acid solution, if there be no irritation. Each successive application, as a rule, causes more smarting. Conjoined with this instillation, a form of manipulation is conducted as follows: The patient, seated in a chair, and the operator standing or sitting behind, two drops of the solution being introduced into each eye, he "places both hands over the closed eyes, so that the tip of each middle finger rests upon the eyeball at its nasal side, the index and ring finger falling into place beside the middle finger. With slight pressure upon the eyeball the three fingers are drawn outward over the eye to the temporal side. This procedure is repeated twenty to thirty times a minute, the stroking being in one direction only, and continued for ten minutes, when a second instillation of two drops into both eyes should be made, manipulation carried on as before for ten minutes; then a third instillation, followed by manipulation for ten minutes. . . . This treatment is to be continued daily for a week, and then the interval between the instillations lengthened to fifteen minutes." The treatment should be continued for three or four months.

Upon his experience in the treatment of twenty-four cases, he formulates the following conclusions: "(1.) Further investigations are necessary before a decided opinion can be expressed as to the result of this treatment of immature cataract. (2.) Immature, uncomplicated cataract can be benefited to the reacquisition of reading power, that is, to good, useful vision. (3.) Incipient cataracts, and those which have but passed into a state of immaturity (!) can be entirely absorbed (?). (4.) This being so, the sooner a cataract comes under treatment, the better the result obtained. (5.) The effect produced . . . is permanent."

---

### FOREIGN BODIES IN THE EYE.

David Webster lays down the following rules: (1.) Always search carefully for foreign bodies on the cornea and on the conjunctiva, in cases of inflammation of one eye coming on suddenly and without other apparent cause. (2.) Remove them, when found, with as little injury as possible to the surrounding parts. (3.) When a foreign body is lodged within the eyeball, especially in the ciliary region, the patient is in danger of losing the fellow-eye by sympathetic inflammation, whether the foreign body is removed or not. The removal of the foreign body greatly lessens such danger. (4.) If the foreign body has already destroyed the sight, the eye should be enucleated without delay. (5.) If sympathetic inflammation sets in, the sooner the eyeball containing the foreign body is enucleated (unless it has servicable vision.—H.) the better will be the patient's chances of retaining useful sight. (6.) If the fellow-eye is attacked with symptoms of severe *sympathetic irritation*, the eye containing the foreign body (if vision in it is lost.—H.) should be enucleated without waiting for actual sympathetic inflammation. (7.) The magnet is serviceable in cases where the foreign body is of attractable material and *can be seen*, and is not firmly embedded in the eye-wall, or encapsuled with organized lymph. (8.) Where the foreign body is small, and its lodging-place is uncertain, the introduction of a magnet into the eyeball is generally to be deprecated. (9.) After the foreign body has been *extracted* from the interior of the eye, the patient should be warned that sympathetic inflammation may occur, and, in such a case, should not be neglected. —*Medical Record*, Nov. 8, 1890.

---

### TREATMENT OF HIGH DEGREES OF MYOPIA BY REMOVAL OF THE LENS.

Dr. Fukala, of Pilsen-Karlsbad, (*Arch. für Ophthal.*, xxxvi., p. 230 ; see, also, *Ophthalmic Review*, October, 1890, p. 304 ; and full translation in *Am. Jour. Ophthalmology*, November, 1890, p. 347,) has, during the past three years, treated twenty-three cases of myopia by removal of the crystalline lens, the object being to diminish the refractive power of the eye by taking away that of the lens, which amounts to 15 to 16 dioptries. The myopia, in these cases, ranged from 11 to 20 dioptries. The oldest patient was 24, the youngest 8. In all, the fundus of the eyes presented a "normal ophthalmoscopic appearance, the crescents ('myopic') being small, or, at most, one-third of a pupillary diameter wide." The state of vision varied from $\frac{1}{25}$ to $\frac{1}{5}$ before treatment, and in every case in which the treatment had been completed (nineteen) it was much improved. The method of treatment was discission of the lens and its absorption. In some instances the discission was repeated as many as fifteen times. In one case, an incision was made in the cornea, and the lens-matter extracted. Some cases were treated without, and some with, iridectomy. The time of treatment varied, in most cases, from three to twelve months. Eyes which were 15 d. to 16 d. myopic were made emmetropic by the treatment, and required no glasses afterwards for distance, while those less myopic needed a corresponding convex glass, and those more myopic, concave glass. The power of accommodation was, of course, lost by taking away the lens, and a glass of 3 d. to 4 d. greater refraction was required for reading in all cases whose myopia was finally changed to emmetropia or hypermetropia, or less than 3 d. to 4 d.

This treatment was pursued under the greatest precautions, and only on young people "with relatively good visual activity, who could read Jæger No. 1 at their *punctura remotum*, and whose fundus presented no choroidal or retinal disease." As the absorption of the lens is not practicable in elderly people, it must be extracted, although the writer had not yet done it. Still, he believes "that with the advances made in the present day, the extraction of the clear lens in high-grade myopia will yield the best results." The advantages which are claimed for this method of treating myopia of high degrees: enlarged retinal images; great improvement of vision for distance; relief of strain and spasm of accommodation, and recession of the far-point, thus reëstablishing binocular vision, which was before impossible on account of the excessive convergence necessary.

